# Microscopic diffusion processes measured in living planarians

**DOI:** 10.1038/s41598-018-22643-9

**Published:** 2018-03-08

**Authors:** Eugene Mamontov

**Affiliations:** Neutron Scattering Division, Oak Ridge National Laboratory, Oak Ridge, TN 37831 USA

## Abstract

Living planarian flatworms were probed using quasielastic neutron scattering to measure, on the pico-to-nanosecond time scale and nanometer length scale, microscopic diffusion of water and cell constituents in the planarians. Measurable microscopic diffusivities were surprisingly well defined in such a complex system as living animals. The overall variation in the microscopic diffusivity of cell constituents was found to be far lower than the variation in the microscopic diffusivity of water in planarians in a temperature range of 284.5 to 304.1 K.

## Introduction

Microscopic diffusion of cell constituents is vital for molecular transport and metabolic activity at the cellular and intra-cellular level. To characterize microscopic diffusion in living animals, here we use quasielastic neutron scattering (QENS), a technique that probes not only the temporal, but also spatial, aspects of biomacromolecular dynamics, which helps elucidate diffusion mechanisms. In the last several years this technique was employed to study intracellular water^1–8^ and intracellular biomacromolecules^9–16^, but a QENS study of a living multicellular organism poses unprecedented challenges. Hydrated encysted brine shrimp eggs investigated using QENS^17^ possibly come closest to the complexity of a living animal, yet they lack intracellular water, being in the state of dormancy. We have identified planarian flatworms as the most suitable candidate for living animal studies. They are three germ-layer acoelomates with solid body and no body cavity, which makes composition of their bodies relatively uniform. They can survive without food for weeks, thus emptiness of their digestive system can be readily achieved by means of withdrawing food. They lack circulatory and respiratory systems, and their metabolism relies on molecular diffusion through the body. Their body shape is conducive to preparation of a relatively thin sample, which is of paramount importance for control of the effects due to multiple neutron scattering in the sample. Finally, they have tremendous ability to stay alive and regenerate into a complete organism if cut into small pieces, and we hypothesized that planarians could be studied even when cut in pieces should our efforts fail to prepare a suitably thin sample using intact living flatworms. Ultimately, cutting was unnecessary, even though the documented inability of planarians to survive even for a few hours in heavy water^18^ necessitated the use of strongly neutron scattering regular water as the host environment for flatworms in the sample.

## Results and Discussion

**Figure 1:**
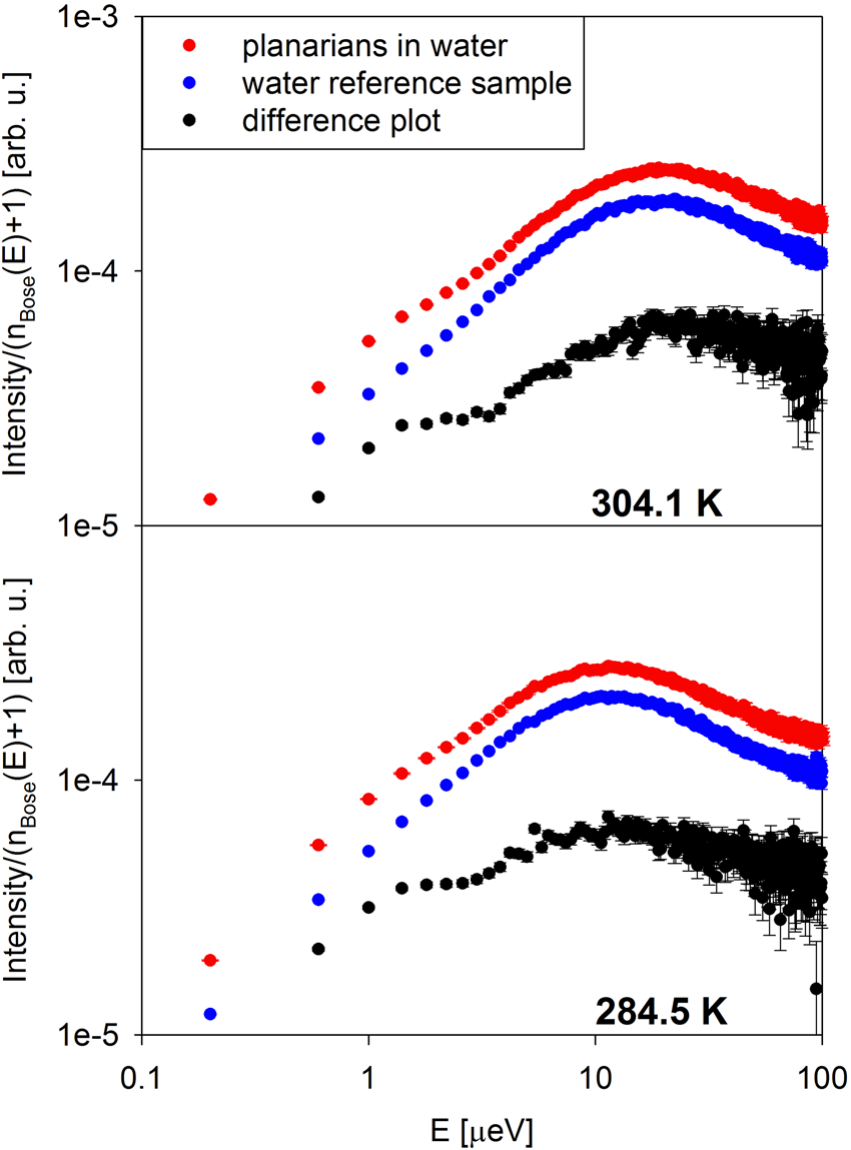
The log-log plot of the neutron scattering signal intensity divided by Bose population factor, I(Q,E)/(n_Bose_(E) + 1), for planarians in water and reference water sample, along with the difference plot. The data collected at the lowest and highest measurement temperatures at Q = 0.3 Å^−1^ are presented.

Figure 1 shows examples of data collected from planarians in water and reference water sample, displayed as I(Q,E)/(n_Bose_(E) + 1), where I(Q,E) is measured neutron scattering signal, n_Bose_(E) = (exp(E/k_B_T)−1)^−1^ is Bose population factor, and k_B_ is Boltzmann’s constant. At sufficiently high energy transfers, where the influence of the spectrometer resolution is relatively small, this data presentation approximates the imaginary part of the dynamic susceptibility. Dynamic susceptibility maxima correspond to the characteristic relaxation times/frequencies in the system, thus allowing illustrative data visualization. In the accessible energy transfer range, there is a single maximum for the H_2_O sample associated with translational water diffusivity. The maximum indicates a characteristic time τ (inversely proportional to the energy position of the peak) it takes for a water molecule moving with a diffusion coefficient D to traverse a distance inversely proportional to the measured momentum transfer, Q, according to the relationship 1/τ = DQ^2^ (for a continuous diffusion process).

The planarians in H_2_O exhibit a very similar peak, evidently originating from water in the sample, but also a visible excess intensity at a low energy transfer of ca. 1 µeV. Considered alone, this does not necessarily indicate the planarian-specific component, unless the excess intensity persists after background subtraction. Indeed, upon subtraction of the H_2_O data from the planarians in H_2_O data, this excess intensity develops into a separate feature, representing the microscopic dynamics in the flatworms, whereas the peak associated with water diffusivity persists, albeit with a lower intensity. Even though the shape of the feature at ca. 1 µeV must be affected by the spectrometer resolution, the data presented in Fig. 1 provide guidance to the I(Q,E) fitting approach. The data from planarians in H_2_O sample (or the difference data obtained by subtracting the H_2_O spectrum) would likely require two fitting components: the broad one, associated with water, and the narrow component that is specific to the planarians.

**Figure 2:**
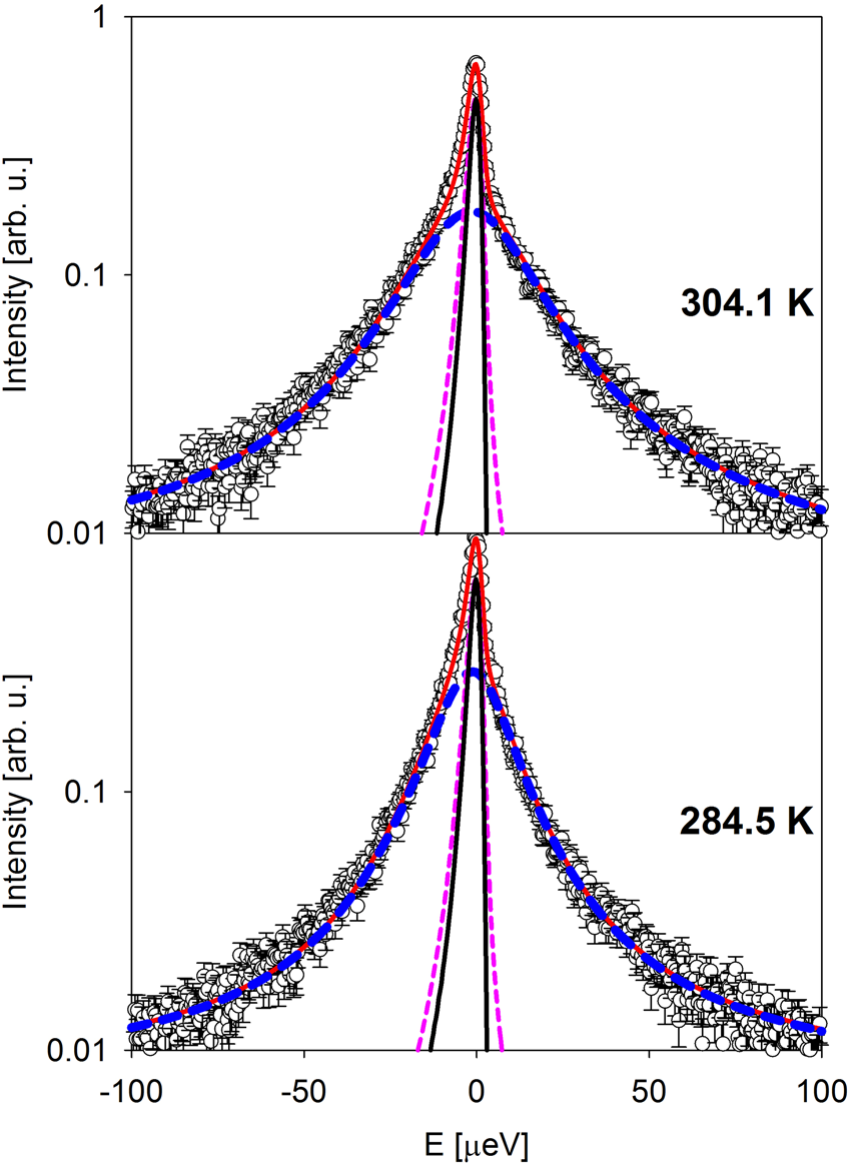
Symbols: I(Q,E), the residual scattering signal (planarians in water sample minus water reference sample) for the data collected at the lowest and highest measurement temperatures at Q = 0.3 Å^−1^, the same data as presented in Fig. 1 by black symbols. Solid red lines: total fit, as described in the text. Long-dashed blue lines: broad fit component with a linear background. Short-dashed pink lines: narrow fit component. The intensity-scaled resolution data collected from the planarians in water sample at 10 K is shown by solid black lines.

All further data analysis was performed on the difference I(Q,E) spectra, as illustrated in Fig. 2 for the same data sets as shown with black symbols in Fig. 1. As demonstrated by the model-independent data presented in Fig. 1, we need to employ a two-component data fit, where the broader component is associated with water. Thus, we used a superposition of a narrow and a broad Lorentzian convolved with the resolution function, R(Q,E), plus a linear background:

1 $$I(Q,E)=R(Q,E)\otimes [x(Q)\frac{{{\rm{\Gamma }}}_{narrow}(Q)}{\pi ({{\rm{\Gamma }}}_{narrow}{(Q)}^{2}+{E}^{2})}+(1-x(Q))\frac{{{\rm{\Gamma }}}_{broad}(Q)}{\pi ({{\rm{\Gamma }}}_{broad}{(Q)}^{2}+{E}^{2})}]+({B}_{1}(Q)E+{B}_{2}(Q))$$

**Figure 3:**
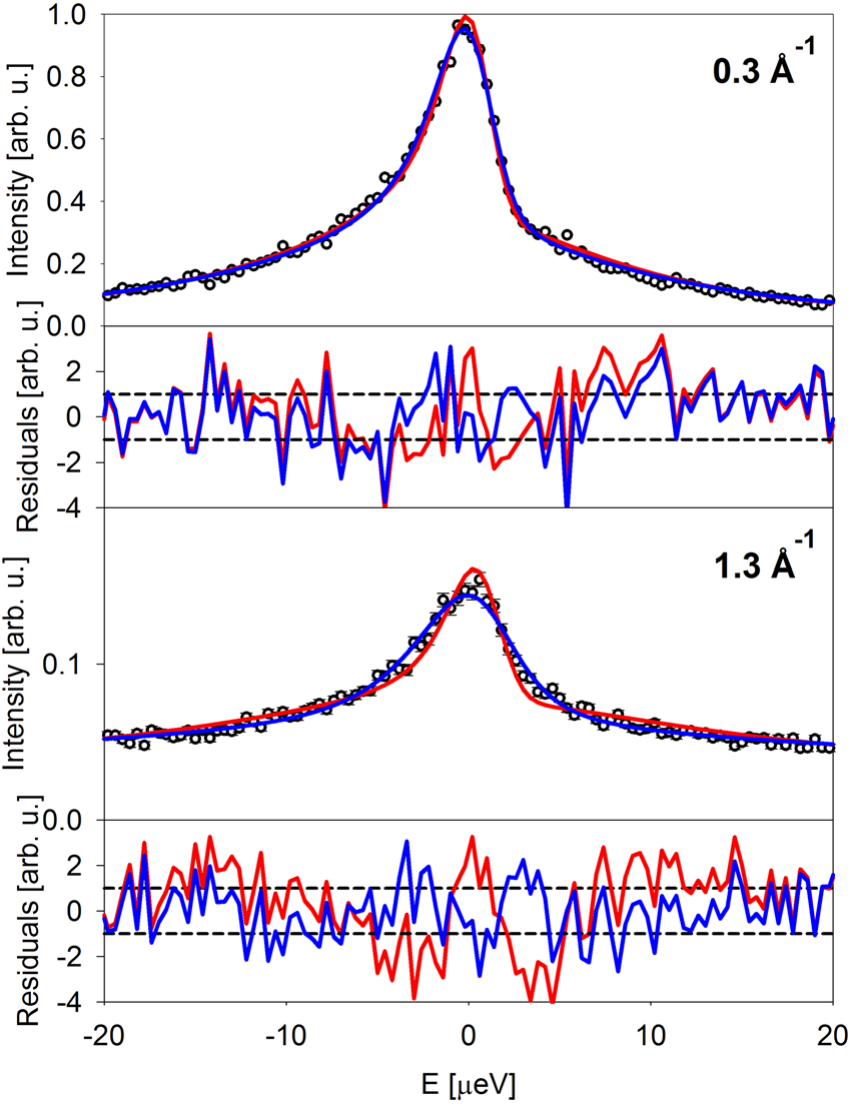
Symbols: I(Q,E), the residual scattering signal (planarians in water sample minus water reference sample) for the data collected at 284.5 K at the lowest and highest measured Q values of 0.3 Å^−1^ and 1.3 Å^−1^. Solid red lines: fits with one-Lorentzian, one delta-function model, as described in the text by Equation 2. Solid blue lines: fits with two-Lorentzian model, as described in the text by Equation 1. The corresponding difference plots (residuals) are also shown with red and blue lines. The data are truncated to ± 20 µeV to emphasize the signal near the elastic line.

The small width of the narrow component demonstrates the necessity of using the sample-specific resolution (see Materials and Methods). Figure 3 shows a comparison of these fit results with an alternative fit approach that assumes a single Lorentzian component and a delta-function component that would account for the scattering from the species whose dynamics might be too slow for the spectrometer resolution:

2 $$I(Q,E)=R(Q,E)\otimes [x(Q)\delta (E)+(1-x(Q))\frac{{\rm{\Gamma }}(Q)}{\pi ({\rm{\Gamma }}{(Q)}^{2}+{E}^{2})}]+({B}_{1}(Q)E+{B}_{2}(Q))$$

This alternative approach produces fits showing a systematic mismatch with the data at low energy transfers, near the elastic line, which quickly worsens at higher Q values. For example, for the 284.5 K data fits presented in Fig. 3, at Q = 0.3 Å^−1^ we obtain the fit agreement factor, χ^2^, of 1.286 and 1.480 using Equations 1 and 2, respectively, whereas at Q = 1.3 Å^−1^ the χ^2^ values become 1.621 and 2.247 for fits with Equations 1 and 2, respectively (the agreement factor *χ*^2^ = Σ(*I*_experiment_ − *I*_model_)^2^/(*N*_observations_ − *N*_parameters_) accounts for possible difference in the number of fit parameters). This comparison confirms the need for a separate narrow fit component to account for the low-energy scattering signal specific to the planarians. All further analysis and discussion pertain to the two-component fits described by Equation 1.

**Figure 4:**
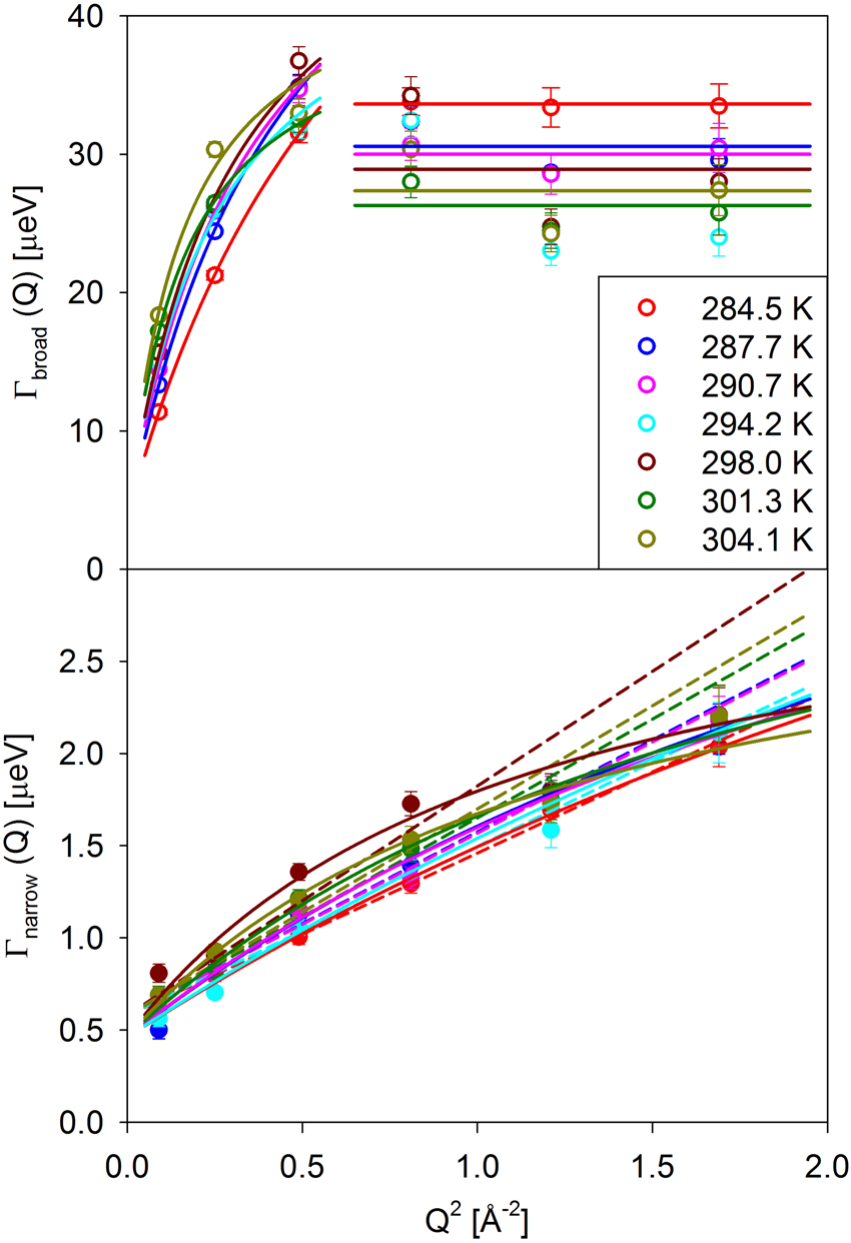
Symbols: narrow (bottom panel) and broad (top panel) fit components associated with the slow and fast diffusivities measured in living planarian flat worms (an example of the data fits is shown in Fig. 2). The components assignment is discussed in the text. Solid lines in the top panel: jump diffusion model fits for the broad component at low Q values and Q-independent fits for the broad component at high Q values. Solid lines in the bottom panel: jump diffusion model fits for the narrow component. Dashed lines in the bottom panel: continuous diffusion model fits for the narrow component.

The Q-dependence of the fitted parameters is presented in Fig. 4. The broad and narrow components are related to water and planarian-specific dynamics, respectively, but more detailed assignment of the components should be guided by the earlier QENS studies of cells^8–15^. Water in planarians could be cytoplasmic, extracellular, or water in bulk form. In a QENS measurement, water in confinement with a characteristic size larger than ca. 10 nm would be indistinguishable from the true bulk water. Furthermore, the exact amount of bulk-like water in living planarians is difficult to quantify. The non-monotonic Q-dependence of the width of the broad component suggests that different water populations give rise to the broad QENS signal at the low and high Q values in the accessible energy transfer range. At the low Q, the signal is dominated by translational diffusivity of bulk water and cytoplasmic water, the diffusion coefficient of which is expected to be similar to that of bulk water^1,4,7,15^. At the higher Q, this signal becomes too broad for the ± 100 µeV measurement range, and the more spatially and dynamically constrained species, such as hydration water molecules in close contact with the cell constituents^8,15^, dominate the measured signal, although cytoplasmic water may still contribute^15^. The width of the measured signal dominated by hydration water, which is expected to be system-specific, exceeds the values for hydration water in prokaryotes *Thermococcus barophilus* and *Thermococcus kodakarensis*^15^, but shows good agreement with the values for eukaryotic human cells of invasive breast carcinoma^8^.While the broad QENS component measured at all Q values largely originates from various water populations, it may also have contribution from the internal dynamics of other cell components, such as cell proteome, the signal from which is comparable in width to the signal from water^15^. In other relevant example, the internal dynamics of alcohol dehydrogenase was measured at about two-thirds of the solvent diffusivity^19^. Besides the too large width of the signal from bulk water, which makes it indistinguishable from the background, the dominance of the hydration water and hydration water-driven proteome dynamics^19^ in the broad QENS signal measured at high Q could be due to the spatially confined character of such dynamics, which thus gives rise to the relatively stronger QENS signal at high Q.

The Γ_broad_(Q) was fitted over the lowest three Q values (see Fig. 4) using a model describing jump diffusion, with diffusion coefficient D and the time between jumps (residence time) τ, and an offset constant B:

3 $${{\rm{\Gamma }}}_{broad}(Q)=B+\frac{\hslash D{Q}^{2}}{1+\tau D{Q}^{2}}$$

In most QENS experiments, when sufficiently thin samples can be prepared, the offset constant is not needed. In the present case, however, the minimum attainable sample thickness is limited by the space needed by the living flatworms and their H_2_O environment (see Materials and Methods), making some multiple scattering of neutrons in the sample unavoidable. Such effects are known to result in some artificial broadening of the QENS signal at low Q values^20^. Appearance of a non-zero offset constant, which nevertheless did not affect the Q-dependence of the QENS signal, has been demonstrated in experiments where multiple scattering was also unavoidable, such as measurements of water at high pressure^21^.

**Figure 5:**
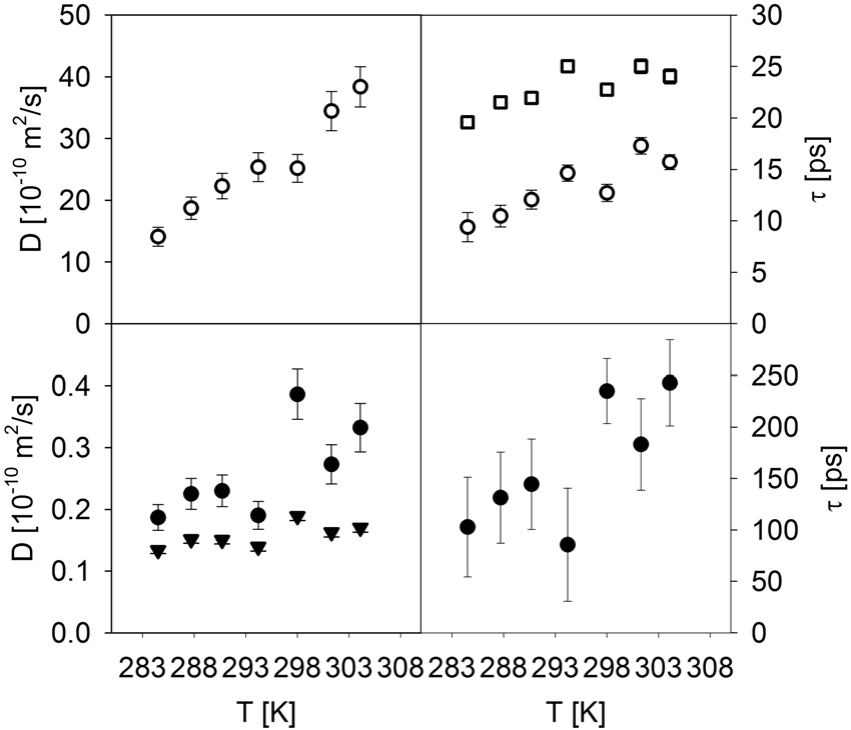
Microscopic dynamics (diffusivity and residence time) in planarian flat worms obtained from the data fits presented in Fig. 4 (top panels, open symbols: broad fit components; bottom panels, filled symbols: narrow fit components). Data fits with jump diffusion model, from which both diffusivity and residence time can be obtained, are shown with circles (open or filled). Data fits with continuous diffusion model, from which only diffusivity can be obtained, are shown with filled triangles. Q-independent data fits, from which only residence time can be obtained, are shown with open squares.

The Q-dependence of the Γ_broad_ was initially analyzed for each temperature individually with B = 0, but, expectedly, yielded artificially high water diffusion coefficients, ranging from (24.2 ± 0.8)×10^−10^ m^2^/s at 284.5 K to (54.4 ± 2.6)×10^−10^ m^2^/s at 304.1 K. Then we repeated the fits using Equation 3 simultaneously for all seven temperatures, with the freely varying parameter B held the same for all temperatures, utilizing this global fitting procedure to facilitate fit convergence. This global fit with B = (3.9 ± 0.7) µeV yielded water diffusion coefficients (top left panel in Fig. 5) ranging from (14.1 ± 1.5)×10^−10^ m^2^/s at 284.5 K to (38.4 ± 3.2)×10^−10^ m^2^/s at 304.1 K, closer to those expected for water at these temperatures^22^. The corresponding residence times are presented in the top right panel in Fig. 5, along with the residence times for hydration water/proteome determined from the Q-independent fits of the broad signal at high Q values, as shown in Fig. 4.

In view of the uncertainty of the exact water populations in the living planarians (bulk vs. cytoplasmic vs. hydration), it is prudent to say, without being more specific, that the broad component in the QENS signal measured at all Q values must be related to the dynamics of water and, possibly, water-driven proteome^19^. On the other hand, the narrow component of the QENS signal represents the microscopic dynamics specific to the cellular constituents of planarians. The Γ_narrow_(Q) was fitted at first using Equation 3 (solid lines in the bottom panel of Fig. 4). This global fit with B = (0.46 ± 0.02) µeV yielded diffusivities and residence times shown in the bottom left and bottom right panels, respectively, of Fig. 5. The error bars on the residence times are rather large, possibly suggesting that the jump diffusion fit model might be too complex for the data. Therefore, we fitted (see dashed lines in the bottom panel of Fig. 4) the Γ_narrow_(Q) using a model describing continuous diffusion with a coefficient D and an offset constant B,

4 $${{\rm{\Gamma }}}_{narrow}(Q)=B+\hslash D{Q}^{2}$$

utilizing a simultaneous fit for all seven temperatures, with the freely varying parameter B held the same for all temperatures. This global fit with B = (0.59 ± 0.01) µeV yielded the diffusivities presented in the bottom left panel of Fig. 5. Similar values of the offset constant B obtained independently in the global fits of the Γ_narrow_(Q) using Equation 3 and Equation 4 suggest that such approach to account for multiple scattering effects is fairly model-independent. The error bars on the diffusivity values become smaller when continuous diffusion model is used.

The microscopic dynamics described by the data in the bottom panels of Fig. 5 pertains to the planarians cells constituents. Biochemical compositions quantified in the literature for some cells such as *Escherichia coli* bacteria and mammalian cells^23,24^ suggest that proteins, DNA/RNA molecules, and lipids/lipopolysaccharides are all present in cells in sufficient quantities to give rise to the sizable scattering signal (aside from the major scattering signal contribution from water). Because the local dynamics of biomacromolecules (e.g., of side groups) is driven by hydration water and is generally too fast to give rise to µeV-range signal at ambient temperature^15,16,19^, one could consider two classes of microscopic processes compatible with the diffusivity values obtained from the Γ_narrow_(Q) (interestingly, both of them are commonly described by continuous, rather than jump, diffusion model). One would be the diffusion of proteins as a whole in the cytoplasmic environment^16,25–32^. However, from the Stokes-Einstein equation, such global diffusivity should be proportional to T/η(T) where η(T) is the medium viscosity. While the water diffusivity (inversely proportional to its viscosity) increased by a factor of 2.7 between 284.5 K and 304.1 K, the concomitant increase in the diffusivities of the cell constituents was merely by a factor of 1.3 for the more plausible continuous diffusion model, or 1.8 for the less plausible jump diffusion model. This deems assignment of the narrow QENS signal predominantly to the global diffusion of intra-cellular biomacromolecules less plausible.

Another class of processes obeying the continuous diffusion law and associated with the measured diffusivity values of 0.1–0.2×10^−10^ m^2^/s is the lateral diffusion of lipids in membranes^33–36^. Although we cannot estimate the relative strength of the QENS signal originating from the lipid assemblies in living planarians, we note that such assemblies may include not only the outer and compartment-separating membranes, but also lipoproteins and intra-cellular lipid droplets in substantial quantitates^37^. Unlike the cell proteome^15,19^ and proteins that are in general coupled to the aqueous solvent^38^, the dynamics and phase transitions in lipid assemblies are decoupled from the solvent^33–36^. Consistent with this, the slow diffusivity measured in planarians varies only by a factor of 1.3–1.8 (depending on the model) between the lowest and highest temperature in the experiment, compared to a factor of 2.7 for water diffusivity.

While largely decoupled from the solvent, the lateral lipid diffusivity may exhibit a sharp increase due to the transition from the gel-like to fluid-like phase. The gel-fluid transition temperature depends on the lipid composition; e.g., 296–297 K in some model membranes^33–36^ and ca. 298 K in low-density lipoproteins^39^ and adipose tissues^40^. To this end, it is interesting to note that the diffusivities in the left bottom panel in Fig. 5, regardless of the fit model chosen (circles or triangles), seem to exhibit systematically higher values above 298 K compared to the lower measurement temperatures. While the effect is rather week and cannot be taken as an evidence of a transition in the dynamics of the cell constituents, it suggests that assignment of the measured slow dynamics to lateral diffusion of lipids at least would not be inconsistent with the well-known phase transition in lipid assemblies. Although the apparent increase in the diffusivity above 298.0 K is not nearly as large as that observed in model membranes of pure phospholipids, various membrane additives are known to reduce, sometimes dramatically, the magnitude of the diffusivity increase at the gel-fluid transition^33–36^.

The rather tightly maintained (compared to water diffusivity) diffusivity of cell constituents may represent a homeostatic parameter resisting changes in the environment. While we have noticed no immediate distress in the control group of planarians exposed to a temperature of 304 K for several hours (similar to the actual experiment), the Planaria Care Sheet^41^ suggests that temperatures higher than 21 to 23 °C (294 to 296 K) may eventually damage planarians. In view of the possible increase in the diffusivity of cell constituent above 298.0 K, we wonder if this empirical knowledge about survival ability of planarians may actually reflect their diminished ability to maintain tightly the diffusivity in their cell constituents at elevated temperatures.

In conclusion, we have found that microscopic diffusivity measured in living animals is surprisingly well defined and can be separated into that of water (hydration, cytoplasmic, and bulk-like) and of the other cell constituents. The latter is maintained much more tightly compared to the former in a temperature range of 284.5 K to 304.1 K, and even more tightly between 284.5 K and 298.0 K.

## Materials and Methods

We used BASIS neutron backscattering spectrometer^42^ at the Spallation Neutron Source, ORNL, operated in a standard mode, with the incident neutron bandwidth centered at 6.40 Å. With the final detected neutron wavelength fixed at 6.267 Å by Si(111) crystal analyzers, this provided a standard range of neutron energy transfers suitable for data analysis between −100 µeV and +100 µeV, while the energy resolution (averaged over all scattering angles) was 3.4 µeV (full width at half maximum). Routine data reduction, including normalization to a flat-plate vanadium standard, was used.

The challenging experiment necessitated adaption of several non-traditional techniques in sample preparation and measurement. Living Brown Planarians specimens were purchased from Carolina Biological Supply Co. To ensure emptiness of their digestive system, the flatworms were not fed for at least a week prior to the experiment. Approximately 30 mid-to large-sized flatworms, ranging in length from a few millimeters to almost a centimeter, were gently loaded using a pipet into a cuvette-type flat-plate geometry aluminum sample holder, 30 mm wide, 70 mm deep, and 1 mm thick, filled with ca. 0.6 g of deionized H_2_O. The resulting total mass of planarian specimens in H_2_O was ca. 0.8 g. Then an aluminum spacer, 30 mm wide, 70 mm tall, and 0.5 mm thick was inserted very slowly into the sample holder, thereby reducing its effective thickness to 0.5 mm. The very slow rate of the spacer insertion was necessary to ensure that the planarians could relocate themselves to the open space in the sample holder. A reference deionized H_2_O sample of ca. 0.6 g was loaded in a similar manner using a separate holder with 0.5 mm thick spacer insert. Thus, the height was at least 40 mm for both the planarians specimens in water and the reference water sample, whereas the nominal size of the neutron beam was 30 mm by 30 mm. The upper portion of the 70 mm tall sample holders thus remained filled with air. In variance with the standard practice, the sample holders were not sealed, and a small gap was maintained using thin spacers between the sample holders and the lids. This was done in order to ensure air exchange between the sample in the cell and the cell’s surrounding to prevent oxygen deprivation to the planarians. The sample holder was attached, by its lid, to a stick inserted into a top-loading close-cycle refrigerator (CCR). Contrary to the standard practice, the CCR was not operated under vacuum or with helium thermal exchange gas, but instead was filled with air, again to prevent oxygen deprivation to the living specimens. Such operation would not be typically possible due to freezing of the air in contact with the cold parts of the CCR. Therefore, the compressor used for operating the CCR’s cold head was turned off, and the CCR was allowed to warm up gradually under helium thermal exchange gas until the cold head temperature exceeded 278 K, when the thermal sensors mounted on the CCR’s inner wall and at the sample position on the stick all showed a similar temperature reading. Then the helium exchange gas was replaced with air and a stick with the attached sample was inserted into the CCR; the unsealed sample holder with the specimens remained open to the surrounding air. From this time on, a heater on the CCR’s inner wall was enabled with a heating ramp rate of 0.03 K/min, while the temperature was read with a sensor at the sample position. The compressor was not operated; therefore, data collection was possible only while the sample was gradually warmed up, with a limited temperature control provided solely by the variable heater output. The data were continuously collected for more than 15 hours on warming up, and subsequently binned into ca. 2 hour-long data sets, to yield the average temperature points separated by 2.8 K to 3.8 K, each point averaged over 2.5 to 4.5 K, namely, 284.5 K (283.3 K–285.8 K), 287.7 K (285.9 K–289.5 K), 290.7 K (289.3 K–292.2 K), 294.2 K (292.0 K–296.5 K), 298.0 K (296.3 K–299.6 K), 301.3 K (299.7 K–303.0 K), and 304.1 K (302.6 K–306.7 K). Then the sample was allowed to warm up to 310 K before the compressor was turned on. In a few minutes, just before the coldest part of the CCR was to drop below 278 K, the air in the CCR was gradually replaced with helium thermal exchange gas. The sample was subsequently cooled down to the baseline temperature of 10 K, and the resolution function was collected from the frozen sample for about 8 hours. Sample freezing for collection of the sample-specific resolution function proved necessary in the data analysis, where one of the fit components was narrow, and the very precise measurement of the resolution function was a must, as oftentimes is the case on the BASIS^42^. Survival of planarians in identical sample holder for a day was thus verified off-line.

The reference H_2_O sample was measured in the same manner, including the CCR warming up with no compressor running and the sample holder open to the surrounding air. This was done to improve the accuracy of the data subtraction, when the water spectra were subtracted from the spectra collected from the planarian specimens in water (measured at the corresponding temperatures). Thus, any increased scattering background due to air in the CCR was cancelled out. The use of H_2_O, necessitated by intolerance of planarians to D_2_O, resulted in a relatively low sample transmission of 75%.

### Data availability

The datasets analyzed during the current study are available from the corresponding author on reasonable request.

## References

[1] Jasnin M, Moulin M, Haertlein M, Zaccai G, Tehei M (2008). Down to atomic-scale intracellular water dynamics. EMBO Rep..

[2] Stadler AM (2008). Cytoplasmic water and hydration layer dynamics in human red blood cells. J. Am. Chem. Soc..

[3] Frolich A (2009). From shell to cell: neutron scattering studies of biological water dynamics and coupling to activity. Faraday Discuss..

[4] Jasnin M, Stadler A, Tehei M, Zaccai G (2010). Specific cellular water dynamics observed *in vivo* by neutron scattering and NMR. Phys. Chem. Chem. Phys..

[5] Natali F (2013). Water Dynamics in Neural Tissue. J. Phys. Soc. Jpn..

[6] Natali F, Gerelli Y, Stelletta C, Peters J (2013). Anomalous Proton Dynamics of Water Molecules in Neural Tissue As Seen By Quasi-Elastic Neutron Scattering. Impact on Medical Imaging Techniques. AIP Conf. Proc..

[7] Liberton M (2013). Organization and flexibility of cyanobacterial thylakoid membranes examined by neutron scattering. J Biol. Chem..

[8] Marques MPM, Batista de Carvalho ALM, Garcia Sakai V, Hatter L, Batista de Carvalho LAE (2017). Intracellular water – an overlooked drug target? Cisplatin impact in cancer cells probed by neutrons. Phys. Chem. Chem. Phys..

[9] Jasnin M, Moulin M, Haertlein M, Zaccai G, Tehei M (2008). *In vivo* measurement of internal and global macromolecular motions in Escherichia coli. Biophys. J..

[10] Stadler AM (2008). Hemoglobin dynamics in red blood cells: correlation to body temperature. Biophys. J..

[11] Marty V (2013). Neutron scattering: a tool to detect *in vivo* thermal stress effects at the molecular dynamics level in micro-organisms. J. R. Soc. Interface.

[12] Peters J (2014). Deep Sea Microbes Probed by Incoherent Neutron Scattering Under High Hydrostatic Pressure. Zeitschrift fur Physikalische Chemie- International Journal of Research in Physical Chemistry & Chemical Physics.

[13] Vauclare P (2015). Molecular adaptation and salt stress response of Halobacterium salinarum cells revealed by neutron spectroscopy. Extremophiles.

[14] Stingaciu LR (2016). Revealing the Dynamics of Thylakoid Membranes in Living Cyanobacterial Cells. Sci. Rep..

[15] Martinez N (2016). High protein flexibility and reduced hydration water dynamics are key pressure adaptive strategies in prokaryotes. Sci. Rep..

[16] Anunciado DB (2017). *In Vivo* Protein Dynamics on the Nanometer Length Scale and Nanosecond Time Scale. J. Phys. Chem. Lett..

[17] Mamontov E (2017). Microscopic diffusion in hydrated encysted eggs of brine shrimp. Biochimica et Biophysica Acta (BBA) - General Subjects.

[18] Lewis GN (1934). The biology of heavy water. Science.

[19] Monkenbusch M (2015). Fast internal dynamics in alcohol dehydrogenase. J. Chem. Phys..

[20] Wuttke J (2000). Multiple-scattering effects on smooth neutron-scattering spectra. Phys Rev. E.

[21] Klotz S, Strassle Th, Bove LE (2013). Quasi-elastic neutron scattering in the multi-GPa range and its application to liquid water. Appl. Phys. Lett..

[22] Mills R (1973). Self-Diffusion in Normal and Heavy Water. J. Phys. Chem..

[23] Cell Biology by the Numbers. What is the Macromolecular Composition of the Cell? http://book.bionumbers.org/what-is-the-macromolecular-composition-of-the-cell/

[24] Delgado, F. F. *et al*. Intracellular Water Exchange for Measuring the Dry Mass, Water Mass and Changes in Chemical Composition of Living Cells. *PloS ONE* **8**, e67590 10.1371/journal.pone.0067590. 10.1371/journal.pone.0067590 PMC3699654 23844039

[25] Perez J, Zanotti JM, Durand D (1999). Evolution of the internal dynamics of two globular proteins from dry powder to solution. Biophys. J..

[26] Gaspar AM, Appavou MS, Busch S, Unruh T, Doster W (2008). Dynamics of well-folded and natively disordered proteins in solution: a time-of-flight neutron scattering study. Eur. Biophys. J. Biophys. Lett..

[27] Roosen-Runge F (2010). Protein diffusion in crowded electrolyte solutions. Biochim. Biophys. Acta.

[28] Roosen-Runge F (2011). Protein self-diffusion in crowded solutions. Proc. Natl. Acad. Sci. USA.

[29] Hennig M (2012). Dynamics of highly concentrated protein solutions around the denaturing transition. Soft Matter.

[30] Grimaldo M, Roosen-Runge F, Zhang FJ, Seydel T, Schreiber F (2014). Diffusion and Dynamics of gamma-Globulin in Crowded Aqueous Solutions. J. Phys. Chem. B.

[31] Grimaldo M (2015). Hierarchical molecular dynamics of bovine serum albumin in concentrated aqueous solution below and above thermal denaturation. Phys. Chem. Chem. Phys..

[32] Grimaldo M (2015). Salt-Induced Universal Slowing Down of the Short-Time Self-Diffusion of a Globular Protein in Aqueous Solution. J. Phys. Chem. Lett..

[33] Sharma VK, Mamontov E, Anunciado DB, O’Neill H, Urban V (2015). Nanoscopic Dynamics of Phospholipid in Unilamellar Vesicles: Effect of Gel to Fluid Phase Transition. J. Phys. Chem. B.

[34] Sharma VK, Mamontov E, Anunciado DB, O’Neill H, Urban V (2015). Effect of antimicrobial peptide on the dynamics of phosphocholine membrane: role of cholesterol and physical state of bilayer. Soft Matter.

[35] Sharma VK, Mamontov E, Tyagi M, Urban VS (2016). Effect of α-Tocopherol on the Microscopic Dynamics of Dimyristoylphosphatidylcholine. J. Phys. Chem. B.

[36] Sharma VK (2016). Dynamical and Phase Behavior of a Phospholipid Membrane Altered by an Antimicrobial Peptide at Low Concentration. J. Phys. Chem. Lett..

[37] Martin S, Parton RG (2006). Lipid droplets: a unified view of a dynamic organelle. *Nature Rev*. Molecular Cell Biol..

[38] Mamontov E, Chu X (2012). Water-protein dynamic coupling and new opportunities for probing it at low to physiological temperatures in aqueous solutions. Phys. Chem. Chem. Phys..

[39] Peters J, Martinez N, Lehofer B, Prassl R (2017). Low-density lipoproteins investigated under high hydrostatic pressure by elastic incoherent neutron scattering. Eur. Phys. J. E.

[40] Sasaki K, Mitsumoto M, Nishiola T, Irie M (2006). Differential scanning calorimetry of porcine adipose tissues. Meat Science.

[41] Planaria. A Carolina^TM^ Care Sheet. https://www.carolina.com/pdf/care-sheets/Planaria-CareSheet.pdf

[42] Mamontov E, Herwig KW (2011). A time-of-flight backscattering spectrometer at the Spallation Neutron Source, BASIS. Rev. Sci. Instrum..

